# Shielding of the geomagnetic field reduces hydrogen peroxide production in human neuroblastoma cell and inhibits the activity of CuZn superoxide dismutase

**DOI:** 10.1007/s13238-017-0403-9

**Published:** 2017-04-26

**Authors:** Hai-tao Zhang, Zi-jian Zhang, Wei-chuan Mo, Ping-dong Hu, Hai-min Ding, Ying Liu, Qian Hua, Rong-qiao He

**Affiliations:** 1 State Key Laboratory of Brain and Cognitive Science, Institute of Biophysics, Chinese Academy of Sciences, Beijing, 100101 China; 2 University of Chinese Academy of Sciences, Beijing, 100049 China; 3 Institute of Beijing Chinese Traditional Medicine, Beijing University of Chinese Medicine, Beijing, 100029 China; 4 CAS Key Laboratory of Mental Health, Institute of Psychology, Beijing, 100101 China

**Keywords:** hypomagnetic field, reactive oxygen species, hydrogen peroxide, superoxide dismutase, oxidative stress

## Abstract

Accumulative evidence has shown the adverse effects of a geomagnetic field shielded condition, so called a hypomagnetic field (HMF), on the metabolic processes and oxidative stress in animals and cells. However, the underlying mechanism remains unclear. In this study, we evaluate the role of HMF on the regulation of cellular reactive oxygen species (ROS) in human neuroblastoma SH-SY5Y cells. We found that HMF exposure led to ROS decrease, and that restoring the decrease by additional H_2_O_2_ rescued the HMF-enhanced cell proliferation. The measurements on ROS related indexes, including total anti-oxidant capacity, H_2_O_2_ and superoxide anion levels, and superoxide dismutase (SOD) activity and expression, indicated that the HMF reduced H_2_O_2_ production and inhibited the activity of CuZn-SOD. Moreover, the HMF accelerated the denaturation of CuZn-SOD as well as enhanced aggregation of CuZn-SOD protein, *in vitro*. Our findings indicate that CuZn-SOD is able to response to the HMF stress and suggest it a mediator of the HMF effect.

**Electronic supplementary material:**

The online version of this article (doi:10.1007/s13238-017-0403-9) contains supplementary material, which is available to authorized users.

## Introduction

Living organisms were exposed in the geomagnetic field (GMF) throughout the evolutionary history. It has been established that that a GMF-shielded condition, so called a hypomagnetic field (HMF) (Mo et al., 2012), adversely affect many aspects of the living system, e.g. embryonic development (Mo et al., 2012; Wan et al., 2014; Osipenko et al., 2008; Fesenko et al., 2010), animal behaviors (Bliss and Heppner, 1976; Prato et al., 2005; Zamoshchina et al., 2012; Mo et al., 2015), and brain function (Zhang et al., 2004; Xiao et al., 2009; Binhi and Sarimov, 2009). Human subjects with short-term exposure to the HMF exhibited enlarged pupil size, increased the number of errors and the task processing time (Binhi and Sarimov, 2009, 2013) and increases capillary circulation and reduction of heart rate (Gurfinkel et al., 2014). Since the HMF is a key environmental factor during long-term and long-distance space mission in outer space, and also in some magnetic shielding conditions on the ground, e.g., the underground bunkers, the inner chamber of a submarine, and the measurement room of magnetoencephalography (MEG) analysis, the biological effect of the HMF should be seriously considered. The adverse physiological effects of the HMF has raised concerns on the potential threat on the health and working capacity of astronauts (Mo et al., 2014). It is necessary to evaluate the effects of HMF exposure, as well as develop effective counteractive strategy against the HMF to protect the health of astronauts and workers who are under occupational HMF exposure. However, the underlying cellular and molecular mechanism of the HMF effects remains unclear, so far.

The redox homeostasis ensures that the cells respond properly to endogenous and exogenous stimuli (Trachootham et al., 2008). Investigating the effect of the HMF on the redox homeostasis will help to elucidate the cellular and molecular responses to the HMF exposure. HMF-induced metabolic changes have been recorded only *in vivo*. The activity of phosphatase in mice decreased after a 18-hour exposure in the HMF (Conley, 1970). One-month stay in the HMF altered the bio-synthetic processes in albino rat (Shust and Kostinik, 1975). Eight-day stay in the HMF led to activation of the adrenal gland function in male albino rat (Shust and Kostinik, 1976). Rabbits subjected to a 2-month HMF exposure since embryogenesis, exhibited disturbed energy metabolism (Kopanev et al., 1979). Five-day HMF exposure leads to increased lipid peroxidation and decreased antioxidant capacity in the internal organs of rat (Babych, 1995, 1996). *In vitro* incubation in the HMF leads to decrease in the activity of aspartate aminotransferase and alanine aminotransferase in human blood sample (Ciorba and Morariu, 2001). According to the radical pair theory, an external magnetic field affects chemical reactions by alternating the electron spin state of a weakly coupled radical pair, which is produced as an intermediate of the electron transport chain reactions (Zhang et al., 2014). Thus, the environmental magnetic fields would probably affect the free radical and intermediates production during the metabolic processes (Fu et al., 2016). Mitochondria is closely related to the regulation of reactive oxygen species (ROS) (He et al., 2016), which has been proposed as the organelle most sensitive to environmental magnetic field (Belyavskaya, 2004). The HMF inhibits mitochondrial function in mouse myocardium and primary muscle cells (Fu et al., 2016; Nepomnyashchikh et al., 1997). By using transcriptome profiling, we found that the differentially expressed genes in the HMF-exposed human neuroblastoma SH-SY5Y cells were involved in the process of metabolism and oxidative stress (Mo et al., 2014). Moreover, Martino and colleagues found that the HMF reduces H_2_O_2_ production in fibrosarcoma HT1080 and pancreatic AsPC-1 cancer cells (Martino and Castello, 2011). Cellular superoxide dismutase (SOD), catalyzes the dismutation of O_2_^.−^ into H_2_O_2_, which is an important antioxidant defense to oxidative stress (Forman, 2016). Therefore, it is worth to investigate the effect of the HMF on the function of key regulators for redox homeostasis for a further understanding on the biomagnetic response.

**Figure 1:**
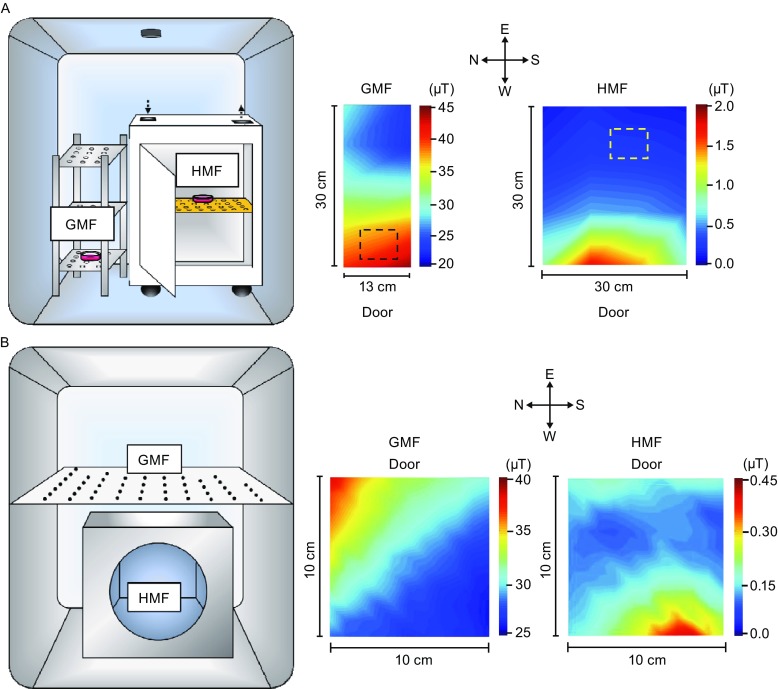
**The experimental set ups for HMF simulation**. (A) The HMF cell incubation system. The permalloy magnetic shielding chambers in the incubators provide the HMF (HMF, <0.2 μT). The GMF control and the HMF-exposed cells were incubated on the bottom floor of the steel shelf (GMF, ~40 μT) and the permalloy chamber, respectively. (B) The HMF enzyme incubation system. The control cells were incubated on the steel shelf (GMF, ~30 μT). The distributions of SMF intensities were shown on the right. Rectangles with dashed lines demarcate the positions for samples

In this study, we aim to evaluate the effect of the HMF on cellular ROS regulation in SH-SY5Y cell when cell proliferation is accelerated (Mo et al., 2013). The level of cellular ROS and its major components H_2_O_2_ and superoxide anion (O_2_^.−^), total antioxidant capacity (Estacio et al., 2015), especially the activity of the key member for ROS/H_2_O_2_ regulation, superoxide dismutase (SOD). We found that the HMF inhibited the activity of CuZn-SOD and that the addition of H_2_O_2_ HMF rescued the HMF-induced accelerated proliferation in SH-SY5Y cell. Our finding presents the evidence that CuZn-SOD is a mediator of the HMF effect, which provides a novel clue to reveal the mechanism of biomagnetic response and to develop the counteractive methods for the HMF effects (Fig. 1).

## Results

### HMF reduces cellular ROS level

**Figure 2:**
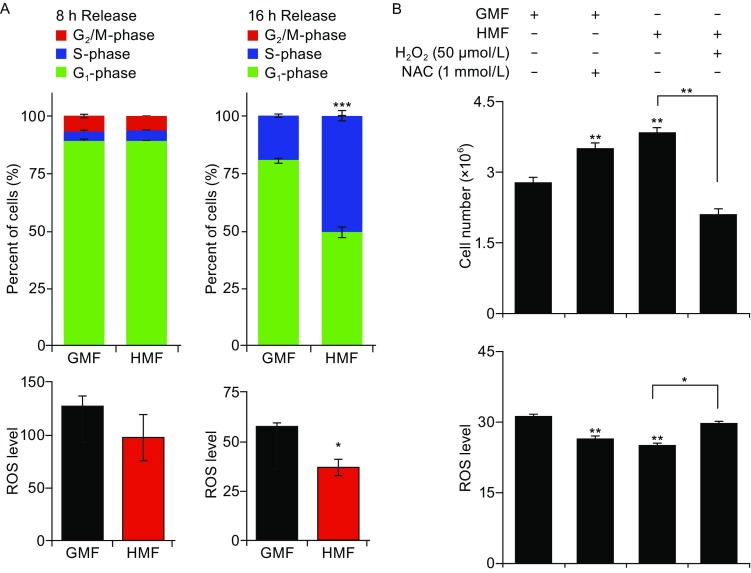
**H**_**2**_ **O**_**2**_ **rescues HMF-accelerated cell proliferation by elevating cellular ROS level.** (A) HMF stimulates G_1_/S transition accompanied with reduction in ROS level. Most G_1_-phase synchronized SH-SY5Y cells remained at G_1_-phase after 8 h releasing and the ROS level was the same between the HMF and GMF group. More HMF-exposed cells entered S-phase, after 16 h releasing; and the ROS level reduced in the HMF group. (B) Reducing the cellular ROS level by 1 mmol/L NAC mimics the HMF-accelerated cell proliferation in the day 2 HMF-exposed non-synchronized cells. Addition of 50 μmol/L H_2_O_2_ in the HMF condition rescues the HMF-induced effects on cellular ROS level and cell proliferation to the GMF-control level. Data were from independent experiments (*n* = 3) and shown as mean ± s.e.m. The *P* values of cell cycle data were calculated using Chi-square test. The data of ROS level and cell counting were calculated using one-way ANOVA by using Bonferroni correction in *post hoc* test. *P < 0.05, **P < 0.01, ***P < 0.001

To exclude the interference effect of un-synchronized cell cycle progression during cell growth, G_1_-phase sychronized SH-SY5Y cells were employed to evaluate the effect of the HMF on ROS regulation. After 8 h releasing in the GMF or HMF, about 90% G_1_-synchronized SH-SY5Y cells were still at G_1_-phase in both groups. The percentages of G_1_-, S-, and G_2_/M-phase cells were the same between the HMF and GMF groups. The ROS level in the HMF-exposed cells showed a reduction tendency but was not significantly different from the GMF-control. After 16 h releasing, the percentage of S-phase cells in the control group was less than 25%; while half of the HMF-exposed cells had entered S-phase (*P* < 0.001; Chi-square test), accompanied with a significant decrease in ROS level, as compared to the control (*P* = 0.035; Student’s *t*-test) (Fig. 2A).

In the non-synchronized cells, the cellular ROS in the day 2 HMF-exposed cells was also lower (*P* = 0.001, HMF vs. GMF; One-way ANOVA Bonferroni *post hoc* test) than that in the control, and the reduction was at the same level with that in cells treated with 1 mmol/L NAC (2-day) (*P* = 0.007, NAC vs. GMF; *P* = 0.059, HMF vs. NAC; One-way ANOVA Bonferroni *post hoc* test), an antioxidant reagent which can reduce cellular ROS and led to ~30% increase in cell proliferation as that in the HMF groups (*P* = 0.022, NAC vs. GMF; *P* = 0.002, HMF vs. GMF; *P* = 0.617, HMF vs. NAC; One-way ANOVA Bonferroni *post hoc* test). Particularly, the HMF-promoted cell proliferation was rescued (*P* = 0.037, H_2_O_2_ vs. GMF; *P* < 0.001, H_2_O_2_ vs. HMF; One-way ANOVA Bonferroni *post hoc* test) by restoring the cellular ROS level (*P* = 0.961, H_2_O_2_ vs. GMF; *P* = 0.007, H_2_O_2_ vs. HMF) with the addition of H_2_O_2_ (50 μmol/L) under the HMF condition (Fig. 2B). No sign of oxidative stress induced lipid peroxidation and cell membrane damage were observed (Fig. S1). The results suggest that the HMF accelerates the proliferation of SH-SY5Y cell by reducing cellular ROS level. But the inhibitory effect of the HMF on ROS level cannot be observed, if cells were pre-incubated in the GMF for 18 h before subjecting to the HMF (Fig. S2).

### HMF inhibits H_2_O_2_ production

**Figure 3:**
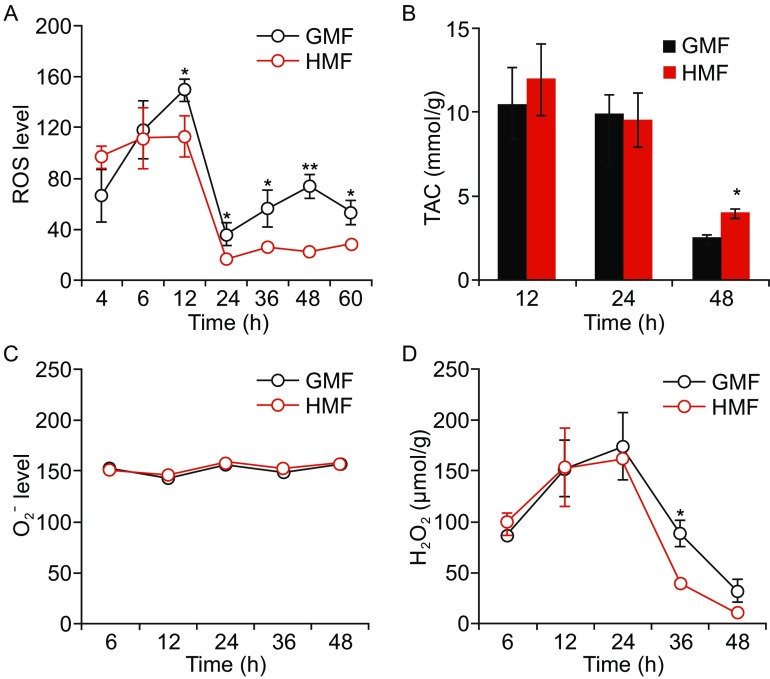
**The dynamic changes of cellular ROS, TAC, H**_**2**_ **O**_**2**_ **, and O**_**2**_^**.−**^. (A) The ROS level in HMF-exposed cells was lower than the control since 12 h. (B) The TAC level in HMF-exposed cells was higher than the control at 48 h. (C) O_2_^.−^ levels in the two groups kept the same level during the exposure. (D) The H_2_O_2_ level in HMF-exposed cells was lower than the control at 36 h. Data were from independent experiments (*n* = 3) and shown as mean ± s.e.m. The *P* values were calculated using one-way ANOVA. *P < 0.05, **P < 0.01

By monitoring the dynamic change in cellular ROS level, we found that both the HMF-exposed and GMF-control cells experienced a ROS increasing period (0–12 h) after seeding, an anti-oxidation period (12–24 h), marked by dramatic decreasing in ROS, and followed with a stabilized low ROS period (24–48 h). The ROS level in the HMF groups was lower than the GMF controls since the anti-oxidation period (Fig. 3A). The TAC of cells in the HMF and GMF groups was at a relatively high level before 24 h and reduced dramatically during the low ROS period (24–48 h), indicating a high-to-low oxidative stress situation corresponding to changes of the ROS level. The TAC of the HMF-exposed cells was significantly higher at 48 h (*P* = 0.014; One-way ANOVA) as compared with the control, which was consistent with the HMF-induced ROS reduction from 24 h to 48 h (Fig. 3B).

The H_2_O_2_ and O_2_^.−^ are the major components of cellular ROS. O_2_^.−^ is spontaneously or enzymatically dismutased to H_2_O_2_ (Rhee et al., 2003). H_2_O_2_ could be subsequently reduced by catalase (CAT) or glutathione peroxidase (GPx). GPx consumes H_2_O_2_ when oxidizing glutathione (GSH) to glutathione disulfide (Droge, 2002). During the 2-day incubation, the O_2_^.−^ levels in the GMF and HMF groups were stabilized at the same level (Fig. 3C). The dynamic changes H_2_O_2_ followed a similar pattern with ROS: a gradual increase before 24 h but dropped down thereafter. The H_2_O_2_ level in the HMF-exposed cells was significantly lower (*P* = 0.029; One-way ANOVA) than that in the GMF control at 36 h (Fig. 3D). The activities of CAT and GPx were not changed by the HMF exposure (Fig. S3), indicating that the HMF did not affect the enzymatic degradation of H_2_O_2_. Thus, the HMF reduces cellular ROS level by inhibiting the production of H_2_O_2_.

### HMF inhibits the activity of CuZn-SOD

**Figure 4:**
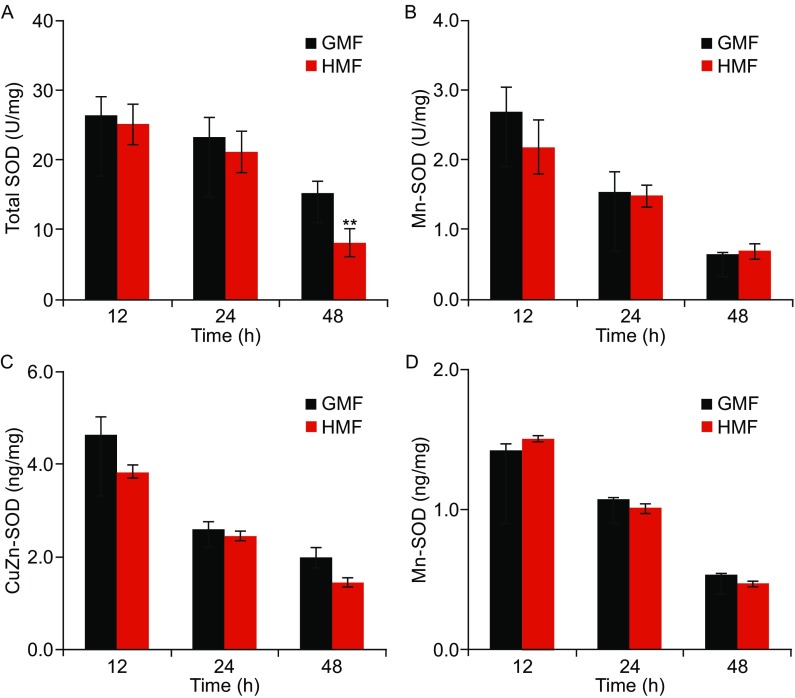
**The HMF inhibits the activity of SOD.** (A) The activity of total SOD was lower in the HMF group than that in the control at 48 h (*n* = 6). (B) The activities of Mn-SOD in the HMF and GMF groups were at the same level along the incubation period (*n* = 6). The expressions of CuZn-SOD (C) and Mn-SOD (D) detected by ELISA were at the same level between the HMF and GMF groups (*n* = 3). *n* is the total number of samples from three independent experiments. Data were shown as mean ± s.e.m. The *P* values were calculated using one-way ANOVA. **P < 0.01

Cellular SODs (CuZn-SOD and Mn-SOD), catalyzes the dismutation of O_2_^.−^ into H_2_O_2_, which is an important antioxidant defense to oxidative stress (Droge, 2002). During the exposure, the activity of total SOD were the same between the GMF and HMF groups and maintained at a relatively high level (~20 U/mg) before 24 h, then decreased from 24 h to 48 h in both groups. The total SOD activity in the HMF group was significantly lowered (*P* = 0.009; One-way ANOVA) at 48 h, as compared with the control (Fig. 4A). While, there was no significant difference in Mn-SOD activity between the GMF and HMF groups during the 48 h incubation (Fig. 4B). The expressions of CuZn-SOD and Mn-SOD proteins was also not changed by the 2-day HMF-exposure (Fig. 4C and 4D). Therefore, we speculated that the HMF inhibits the activity of cellular CuZn-SOD.

**Figure 5:**
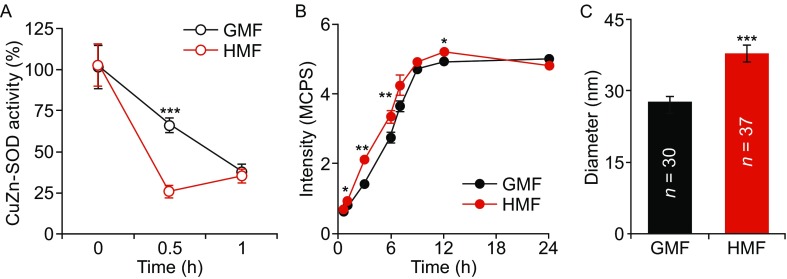
**The HMF inhibits the activity of CuZn-SOD by inducing protein aggregation.** (A) The activity of CuZn-SOD decreased faster in the HMF than the GMF (*n* = 9). (B) Rayleigh scattering assay showed that larger protein aggregates were formed in the HMF (*n* = 3). (C) AFM indicated that the size of protein aggregation was larger after 30 min HMF exposure (*n* = 37) than the controls (*n* = 30). CuZn-SOD was dissolved in water. *n* is the total number of samples from three independent experiments. Data were shown as mean ± s.e.m. The *P* values were calculated using one-way ANOVA. *P < 0.05, **P < 0.01, ***P < 0.001

To confirmed the inhibitory effect of the HMF on CuZn-SOD, CuZn-SOD (0.88 ug/mL) solution was incubated in the GMF and HMF at 37°C. As the activity of CuZn-SOD dropped down very fast in the solution during thermal denaturation (Fig. S4), we measured the effect of HMF exposure on the denaturation of CuZn-SOD at 0 h, 0.5 h, and 1 h. The activity of CuZn-SOD reduced much faster (*P* < 0.001; One-way ANOVA) in the HMF than in the GMF (Fig. 5A). Only 25% enzyme activity retained after a 30 min-HMF exposure. Meanwhile, 60% activity of CuZn-SOD maintained in the GMF. CD spectrum assay showed that the secondary structure of CuZn-SOD was not changed by 0.5 h HMF-exposure (Fig. S5A). The intrinsic fluorescence of the HMF-exposed and GMF-control samples (30 min) was also the same, indicating the HMF did not change the conformation of the CuZn-SOD (Fig. S5B). As protein aggregates during denaturation (Zhao et al., 1998), we monitored the progress of protein aggregation in CuZn-SOD solution by using Rayleigh scattering. It was observed that the particle size increased along with the decrease in CuZn-SOD activity and the increase in particle size was significantly faster than that in the HMF (Fig. 5B). AFM images showed that larger protein aggregates (38.0 ± 1.6 nm; *P* < 0.001, One-way ANOVA) were formed in the CuZn-SOD solution after 30 min HMF-exposure, when compared with the GMF control (27.8 ± 1.1 nm) (Fig. 5C). The results above suggest that HMF inhibits the activity of CuZn-SOD by accelerating its denaturation.

## Discussion

In this work, we found that the redox homeostasis of SH-SY5Y cells can response to the magnetic field, and HMF exposure accelerates the cell proliferation by decreasing the cellular ROS level. Redox homeostasis plays an important role in aging and diseases (Forman, 2016). Excessive ROS induced oxidative stress is a frequent complication in disease conditions (Droge, 2002). Aberrant or elevated levels of ROS can mediate deleterious cellular effects, including neuronal toxicity and degeneration observed in the etiology of a number of pathological conditions (Trachootham et al., 2008). While, accumulating evidence has shown that reduction in cellular ROS could stimulate cancer cell proliferation (Song et al., 2014). Our data shows that the ROS level in SH-SY5Y cells decreases in the HMF and no sign of oxidative stress-induced lipid peroxidation and cell membrane damage were observed, which eventually lead to increase in cell proliferation. Decrease in ROS level under low level magnetic field was also observed by Martino and colleagues in several cancer cell lines (Martino and Castello, 2011). The HMF-induced neural stem cell proliferation would probably also results from the HMF-induced ROS reduction (Fu et al., 2016). However, previous reports have shown that HMF exposure could lead to increase in oxidative stress in animals (Babych, 1995, 1996). The increase in cellular ROS level were also detected in the HMF-exposed primary cultures of mouse muscle cell (Fu et al., 2016). Although we did not observe increase in serum H_2_O_2_ level in mice after 30-day HMF exposure, decrease in general activity and disorder in circadian drinking rhythm were recorded (Mo et al., 2015). Therefore, we speculate that a HMF condition is probably not a stress factor for cancer cells but might raise oxidative stress in differentiated/somatic cells. In addition, it has been reported that exposure to a strong SMF (2.3 mT) increases ROS production in SH-SY5Y cells (Calabro et al., 2013). The ROS level may serve as a characteristic index for the cellular response to environmental magnetic exposure.

We also found that CuZn-SOD may play as a mediator of the HMF effect, as HMF represses the activity of CuZn-SOD but not Mn-SOD, and enhances aggregation of CuZn-SOD protein. CuZn-SOD (SOD1) is a soluble cytoplasmic and mitochondrial intermembrane space protein (Li et al., 2013). Mn SOD is located in the mitochondrial matrix (Zhu and Scandalios, 1995). It has been shown that mitochondrion is sensitive to the HMF (Fu et al., 2016; Belyavskaya, 2004; Nepomnyashchikh et al., 1997). Based on our data, no significant difference was detected on the expression and activity of Mn-SOD between the GMF and HMF groups; while the denaturation of CuZn-SOD was directly accelerated by the HMF in the solution, a cell-free condition. Thus, the intermembrane mitochondrial CuZn-SOD would probably contribute to the HMF-inhibited mitochondrial function. The mature CuZn-SOD protein is highly stable, but unstable when in its metal-free and disulfide-reduced forms. The loss of metal ions results in increased CuZn-SOD aggregation (Estacio et al., 2015). In disease models, low metallation is observed for insoluble CuZn-SOD (Khare et al., 2004). The surface-exposed reduced cysteines could participate in disulfide crosslinking and, thus, aggregation (Estacio et al., 2015). Our data showed that the HMF did not change the secondary structure and conformation of CuZn-SOD but accelerate the aggregation of CuZn-SOD in the solution. As the cellular plasma of the HMF-exposed cell exhibited a less oxidative state, we speculate that the elimination of the GMF would weaken the binding of metal ions with the enzyme or facilitate the reduction of the cysteine.

We noticed that the early 18 h incubation in the HMF was critical to the inhibitory effect of the HMF in ROS production (Fig. S1). In this study, SH-SY5Y cells were transferred to the HMF condition immediately after seeding, in the form of cell suspension. The signals generated during cell attachment have been shown to be strongly affected by integrin-triggered production of ROS from several sources. ROS were reported to have important roles in integrin-mediated attachment, spreading, and the associated changes in the cytoskeleton (Zeller et al., 2013). Exogenous administration of hydrogen peroxide (0.5 mmol/L) was recently shown to promote actin polymerization in the lamellipodia of PtK1 epithelial cells (Taulet et al., 2012). The observed ROS reduction in our study is consistent with our previous report that HMF inhibits actin assembly (Mo et al., 2016). The 18 h pre-incubation in the GMF probably bypass the high ROS and Cu/Zn SOD level period which is sensitive to HMF, thereafter, provide a protective effect against the disturbance of the HMF on ROS production, as well as the relevant actin assembly and proliferation. However, as the current evidence shown that actin assembly (cell motility) gave a quick response to HMF no later than the ROS level, further evidence is necessary to tell whether the actin response plays a role in the effect of HMF on ROS level and cell proliferation.

## Conclusion

In this paper, we foundd that HMF accelerates the proliferation of SH-SY5Y cell by decreasing the cellular ROS level, and that the anti-oxidation effect is caused by the reduction in H_2_O_2_ production, which is accompanied with the inhibition in CuZn-SOD activity. CuZn-SOD plays as a mediator of the HMF effect and would be a potential target for the development of counteractive method against the HMF effect.

## Materials and Methods

### The HMF conditions

The HMF condition for cell culture was provided by a permalloy magnetic shielding chamber (MSC) as described previously (Mo et al., 2013, 2016). The MSC were settled in a Hera240i incubator (Thermo Fisher Scientific, Waltham, MA, USA). The incubator was set as 95% relative humidity, 37°C and 5% CO_2_ concentration. The residual static magnetic field (SMF) inside the MSC was < 0.2 μT. The GMF control samples were cultured on a plastic shelf outside the MSC with a local SMF of 39.4 ± 3.6 μT (Fig. 1A). The ambient alternating magnetic field (AMF) was 22.5 ± 0.0 nT in the MSC (without dominant frequency) and 77.4 ± 1.2 nT on the GMF control shelf (dominant at 50 Hz).

The HMF condition for CuZn-SOD incubation was provided by another permalloy MSC constructed by the National Space Science Center (NSSC) of Chinese Academy of Sciences (Beijing, China), settled in an HWS-250 thermostatic incubator (Shanghai Sumsung Experimental Instrument, Shanghai, China). The incubator was set at temperature 37°C without CO_2_ input, as the CO_2_ for cell culture (5%) will affect the chemical reaction in the cell-free system. The residual SMF inside the MSC was < 0.2 μT. The GMF control samples were cultured on a stainless steel shelf on top of the MSC with a local SMF of 29.9 ± 2.85 μT (Fig. 1B). The ambient AMF was 22.0 ± 1.0 nT in the MSC (without dominant frequency) and 2.74 ± 0.07 μT on the GMF control shelf (dominant at 50 Hz).

The SMFs were measured by a Fluxgate Magnetometer (NSSC, Beijing, China). The AMFs were measured with a CCG-1000 induction alternative magnetometer (National Institute of Metrology, Beijing, China). The magnetic field conditions were listed in Table 1.

**Table 1:** **The magnetic field conditions** ^**a**^

		**GMF-a** ^**c**^	**HMF-a** ^**c**^	**GMF-b** ^**d**^	**HMF-b** ^**d**^
**SMF**	|B|^b^ (μT)	39.4 ± 3.6	0.19 ± 0.08	29.9 ± 2.85	0.14 ± 0.07
**AMF**	|B| (nT)	77.4 ± 1.2	22.5 ± 0.0	2,740 ± 68	22.0 ± 1.0
	*Dominant f* (Hz)	50	/	50	/
	*f* range (Hz)	50–1000	2,200–2,700	50–55	2,300–2,900

^a^Data were shown as mean ± s.d

^b^The vector sum of the magnetic field in three directions

^c^The HMF system for cell culture

^d^The HMF system for enzyme incubation

### Cell culture

Human neuroblastoma SH-SY5Y cells (China Cell Resource Confederation, Beijing, China) were maintained in DMEM (High D-glucose) (Gibco/Invitrogen, Grand Island, NY, USA) supplemented with 10% (*v*/*v*) fetal bovine serum (FBS; PAA Laboratories, Pasching, Austria), 100 unit/mL penicillin and 100 μg/mL streptomycin (Gibco/Invitrogen, USA) as monolayer in petri dishes (NEST Biotechnology, Wuxi, Jiangsu, China) at 37°C and 5% CO_2_ with > 95% relative humidity as described previously (Mo et al., 2013, 2016). Cells were passaged every two days. Cell numbers were counted using a Countess automated cell counter (Life technologies/Invitrogen, USA).

### Cell cycle assay

SH-SY5Y cells were synchronized at the G_1_-phase by serum starvation (DMEM with 1% FBS) and cell cycle progression was measured with flowcytometry as described in Mo et al. (2013). The starved, G_1_-arrested cells were harvested by trypsinization, seeded into 60 mm petri dishes at the density of 3.0 × 10^4^ cells/cm^2^ and cultured in the release medium (DMEM with 20% FBS). Cells were fixed in 75% ice-cold ethanol, and re-suspended in 1 mL phosphate buffered saline (PBS, pH = 7.2) containing 50 μg/mL propidium iodide (PI; Sigma-Aldrich, St. Louis, MO, USA) and 1 mg/mL RNase A (Sigma-Aldrich, USA). The DNA content was monitored with a Becton Dickinson FACSCalibur flow cytometer (BD Bioscience, Franklin Lakes, NJ, USA). Cell cycle was analyzed with ModFit LT software (Verity Software House, Topsham, ME, USA).

### ROS assay

The cellular ROS level was determined with the reagent 2’-7’-dichlorodihydrofluorescein diacetate (DCFDA) (Beyotime, Jiangsu, China). In brief, cells were seeded into 60 mm petri dishes at the density of 3.0 × 10^4^ cells/cm^2^. After 48 h incubation in the HMF or GMF condition, cells were loaded with 10 μmol/L DCFDA for 30 min at 37°C. After trypsinization, the fluorescence intensity of the harvested cells was measured by the flow cytometer (BD Bioscience, USA) with excitation at 488 nm and emission at 525 nm. Data were analyzed by the Cell Quest Pro software (BD Bioscience, USA). The geometric mean of the fluorescence value was used for mean comparison. Each measurement was performed in triplets. Antioxidant reagent, N-acety-L-lysine (Jelenkovic et al., 2006) (Beyotime, China), and H_2_O_2_ (Beijing chemical works, Beijing, China) were used to interfere with the cellular ROS level.

### TAC assay

The TAC was measured using a TAC assay kit with the 2, 2’-azino-bis(3-ethylbenzthiazoline-6-sulfonic acid) (ABTS) method (Beyotime, China). Cells were seeded in 60 mm petri dishes as described above. Cells were harvested and homogenized with the lysis buffer provided by the kit at 4°C. After 10 min centrifugation at 12,000 rpm (Medifriger-BL-S, P-Selecta, Barcelona, Spain) at 4°C, the supernatants were collected for the test. The protein concentration was determined by a bicinchoninic acid (BCA) protein assay kit according to the manufacturer’s instruction (Pierce/Thermo Fisher Scientific, USA). 10 μL cell lysate was transferred to a 96-well plate and mixed with 200 μL ABTS working solution. After 5 min room temperature (RT) incubation, absorbance at 734 nm was measured by using a microplate reader (Biorad, Hercules, CA, USA). The TAC of each sample was calculated by the prepared standard curve and normalized by protein concentration. Each measurement was performed in triplets.

### **O**_**2**_^.−^**assay**

The cellular O_2_^.−^ level was detected with the fluorescent probe dihydroethidium (DHE) (Beyotime, China). Cells were seeded in 60 mm petri dishes as described above. In brief, cells were loaded with 1 μmol/L DHE for 30 min at 37°C. After trypsinization, the fluorescence intensity of the harvested cells was measured by the flow cytometer (BD Bioscience, USA) with excitation at 535 nm and emission at 610 nm. Data were analyzed by the Cell Quest Pro software (BD Bioscience, USA). The geometric mean of the fluorescence value was used for mean comparison. Each measurement was performed in triplets.

### H_2_O_2_ assay

The cellular H_2_O_2_ level was measured using a H_2_O_2_ assay kit (Beyotime, China). Cells were seeded in 60 mm petri dishes as described above. Cells samples were prepared as described above. 50 μL sample was mixed with 100 μL working solution and incubated for 30 min at RT. The absorbance at 560 nm was measured by using a microplate reader (Biorad, USA). The H_2_O_2_ concentration was calculated by the prepared standard curve and normalized by protein concentration. Each measurement was performed in triplets.

### SOD assay

The activity of total SOD (CuZn/Mn) in cells or SOD solution was measured with a total SOD assay kit (Beyotime, China). Cells were seeded in 60 mm petri dishes as described above. Cell lysates were prepared as described above. Bovine erythrocytes CuZn-SOD powder was purchased from (Sigma, USA). 20 μL cell lysate was transferred to a 96-well plate and mixed with 160 μL WST-1 working solution and 20 μL enzyme working solution. After 20 min incubation at 37°C, the absorbance at 450 nm was measured using a microplate reader (Biorad, USA). The activity of Mn-SOD in cells was measured with a Mn-SOD assay kit (Beyotime, China). Dilute the lysate samples with CuZn-SOD inhibitor A at 24:1 and incubate the mixture at 37°C for 1 h. Next, dilute the mixture with CuZn-SOD inhibitor B at 25:1 and incubate the mixture at 37°C for 15 min. 20 μL mixture was transferred to a 96-well plate and mixed with 160 μL WST-8 working solution and 20 μL enzyme initiation solution. After 30 min incubation at 37°C, the absorbance at 450 nm was measured using a microplate reader (Biorad, USA).The SOD activities were calculated by prepared standard curves. The final data of SOD activities were normalized by protein concentration. Each measurement was performed in triplets.

### Enzyme-linked immune-sorbent assay (ELISA)

The cellular CuZn-SOD and Mn-SOD contents were measured with SOD1 and SOD2 ELISA kit (Cusabio, Wuhan, China), respectively. Cells were seeded in 60 mm petri dishes as described above. Cells were harvested after 12 h, 24 h, and 48 h growth in the HMF and GMF. After twice wash with PBS, cells were scraped into 500 μL cold PBS. After mechanical cell disrupting, total protein samples were send for centrifugation at 11,000 ×*g* for 15 min. Protein concentration was determined as described above. All of the ELISA procedures strictly followed the manufacturer’s instructions. In brief, protein samples were pre-incubated with the adhesive strip provided 2 h at 37°C. After removing the liquid of each well, add 100 µL of biotin-antibody working solution to each well and incubate for 1 h at 37°C. After thrice washing with 350 µL washing buffer, invert the plate and blot it against clean paper towels. Add 100 µL of HRP-avidin working solution to each well and incubate for 1 h at 37°C. After thrice washing with 350 µL washing buffer, add 90 µL of TMB substrate to each well and incubate for 30 min at 37°C in the dark. Add 50 µL of stop solution to each well and determine the absorbance at 450 nm using a microplate reader (Biorad, USA). The content of SOD proteins was calculated by a prepared standard curve. The final data of SOD expressions were normalized by protein concentration. Each measurement was performed in triplets.

### Rayleigh scattering

The protein aggregation in the SOD solution was monitored by Rayleigh scattering by using the FluoroMax-4 Spectrofluorometer (Horiba scientific, Kyoto, Japan), as described in Liu et al. (2009). In brief, CuZn-SOD water solution (0.88 μg/mL) was incubated in the HMF and GMF at 37°C for 24 h. Aliquots of the HMF-exposed and GMF-control solutions were collected at time points (0.5 h, 1 h, 3 h, 6 h, 7 h. 9 h, 12 h, and 24 h). The input light was set at 280 nm and the intensity of the scattering light at 559 nm was measured in the unit of million count per second (MCPS). Each measurement was performed in triplets.

### Atomic force microscopy (AFM)

The size of SOD enzyme aggregation was measured by using AMF (Mutiplemode-I, Digital Instruments, USA). CuZn-SOD (0.88 μg/mL) solutions were incubated in the GMF and HMF at 37°C for 0.5 h and then sent for AMF measurement. All solutions used were filtered through a 0.22 µm filter (Millipore, Billerica, MA, USA). Aliquots (10 µL) were allowed to adsorb onto the mica and were kept at RT for 5 min before observation. The particle size was measured as described previously (Chen et al., 2010).

### Statistical methods

Data were shown as mean ± s.e.m. of at least three independent experiments. The normality of the data was examined by Shapiro-Wilk test. One way ANOVA were applied for mean comparison. For multiple comparison, Bonferroni *post hoc* test was used after one-way ANOVA. The cell cycle data were analyzed with chi-square test. Significance was accepted at *P* < 0.05.

## Electronic supplementary material

Below is the link to the electronic supplementary material.

Supplementary material 1 (PDF 378 kb)
