# Supplementary material for: Shielding of the geomagnetic field reduces hydrogen peroxide production in human neuroblastoma cell and inhibits the activity of CuZn superoxide dismutase
Source: Protein Cell. 2017 Apr 26;8(7):527–37. doi: 10.1007/s13238-017-0403-9 (PMC5498340; doi:10.1007/s13238-017-0403-9)
Supplement: Supplementary file 1 — Supplementary material 1 (PDF 378 kb) [file 13238_2017_403_MOESM1_ESM.pdf]

Supplementary Materials

Materials and Methods

Methane dicarboxylic aldehyde (MDA) assay

MDA is the end product of lipid peroxidation and commonly considered as an indicator of oxidative stress. Cellular MDA levels were measured with the lipid peroxidation MDA assay Kit (Beyotime, Jiangsu, China) by the thiobarbituric acid (TBA) method. Cells were seeded into 60 mm petri dishes at the density of 3.0×10^4 cells/cm^2. Cell lysates and protein concentration were prepared as described in the material and methods for TAC assay. Absorbance at 532 nm was measured by using a microplate reader (Bio-Rad, USA). The final data of MDA levels were normalized by protein concentration. Each measurement was performed in triplets.

Lactate dehydrogenase (LDH) assay

The activity of LDH released in the supernatant medium was measured with a LDH cytotoxicity assay kit (Cayman Chemical Company, Ann Arbor, MI, USA). The activity of LDH was determined by the conversion of tetrazolium salt into a red formazon product. Cells were seeded in 60 mm petri dishes as described above. In brief, 100 μL medium was transferred to a new 96-well plate and mixed with 100 μL reaction solution. After incubating the plate with gentle shaking for 30 minutes at RT, absorbance at 490 nm was measured using a

microplate reader (Biorad, USA). The concentration of the released LDH was calculated by the prepared LDH standard curve. Each measurement was performed in triplets.

Catalase (CAT) assay

The activity of cellular CAT was measured with a catalase assay kit (Beyotime, China). Cells were seeded in 60 mm petri dishes as described above. Cell lysates and protein concentration were prepared as described above. Cell lysate was mixed with buffer solution and 250 mM H_2O_2. The mixture was incubated at 25 ℃ for 5 min. 50 μL sample solution was mixed with 450 μL catalase stopping solution with vortex to generate a 500 μL stopped sample. After mixing 40 μL buffer solution with 10 μL stopped sample, transfer 10 μL of the mixture to the 96-well plate and incubate with 200 μL working solution at 25 ℃ for 30 min. The absorbance at 520 nm was measured using a microplate reader (Biorad, USA). The activity of CAT was calculated by a prepared standard curve. The final data of CAT activity was normalized by protein concentration. Each measurement was performed in triplets.

Glutathione peroxidase (GPx) assays

The cellular GPx activity was measured with a cellular GPx assay kit (Beyotime, China). Cells were seeded in 60 mm petri dishes as described above. Cell lysates and protein concentration were prepared as described

above. Mix 10 μL GPx working solution, 176 μL assay buffer and 4 μL H_2O_2 (15 mM) with 10 μL cell lysate. The absorbance at 340 nm (A_{340}) of the mixture was measure every 30 s for 3 min at 25 ℃ by a microplate reader (Biorad, USA). The GPx activity was determined by the rate of A_{340} change (ΔA340/min). The final data of GSH reductase activity were normalized by protein concentration. Each measurement was performed in triplets.

Circular dichroism (CD) spectrum assay

CD measurements were performed using a Chirascan plus (Applied Photophysics, Leatherhead, Surrey, UK) at RT. The CD scans were recorded within a wavelength range of 180 to 260 nm. All measurements were performed in a cuvette with a volume of 200 μL in ddH_2O. Individual titrations were performed with 0.176mg/ml CuZn-SOD in a ddH_2O incubated in GMF and HMF at 37° C for 0.5 h. ddH_2O was used as the blank. The spectra were measured based on an average of three runs.

Fluorescence spectra assay

CuZn-SOD (sigma, USA) solution (0.88 μg/ml) were prepared by diluting the stock solution with ddH_2O. The solution was incubated in the GMF and HMF condition at 37℃ for 30 min. The intrinsic fluorescence spectra of the solutions were determined by using Multiscan Spectrum (infinite M200PRO, TECAN, USA). The excitation wavelength was set at 280 nm and the emission light

were scanned from 180-300 nm at 1 nm wavelength step.

Statistical methods

Data were shown as means ± s.e.m. of at least three independent experiments. The normality of the data were examined by Kolmogorov-Smirnov test. One way ANOVA were applied for mean comparison. For multiple comparison, Bonferroni post hoc test was used after one-way ANOVA. Significance was accepted at $p < 0.05$.

Figures

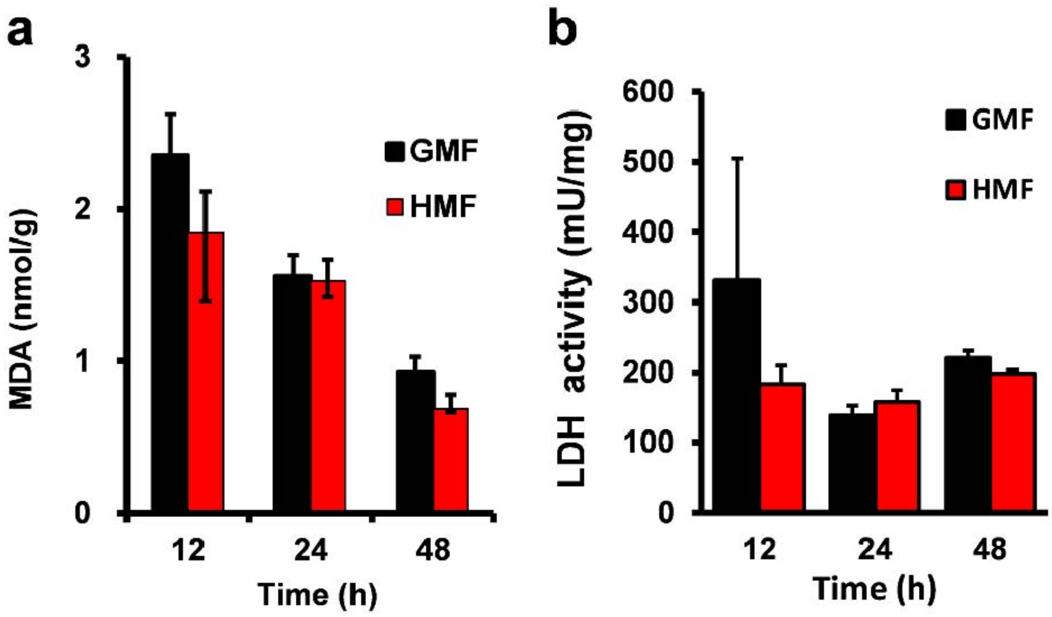

Supplementary Figure 1. The HMF induces no lipid peroxidation and membrane damage. (**a**) MDA assay. (**b**) LDH assay. Data were from independent experiments (n=3) and shown as mean ± s.e.m.

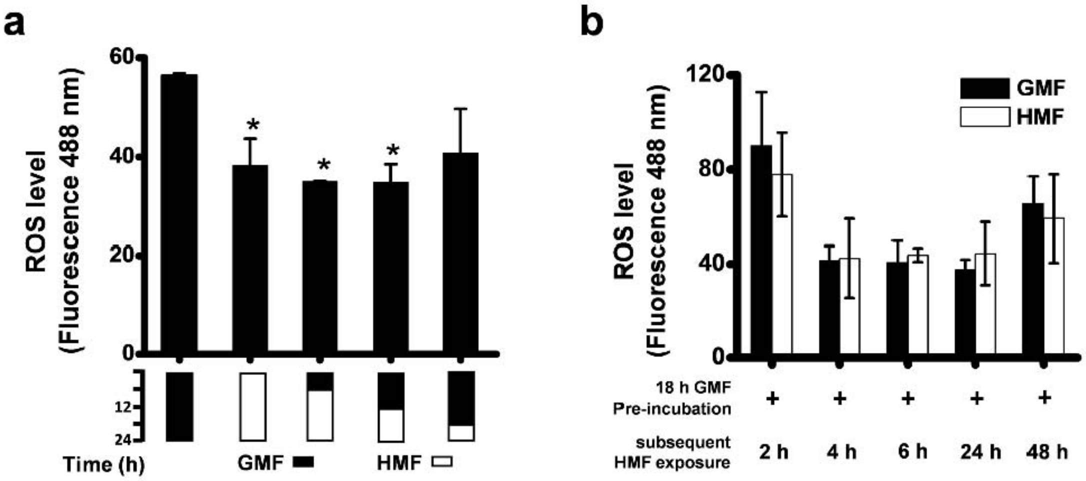

Supplementary Figure 2. The early HMF exposure period attributes to the HMF's inhibitory effect on ROS. The effective period of the HMF exposure on ROS is before 18 h (a). HMF exposure after 18 h in the GMF cannot affect the cellular level (b). Data were from independent experiments (n=3) and shown as mean ± s.e.m. Data were calculated using ANOVA. *P<0.05.

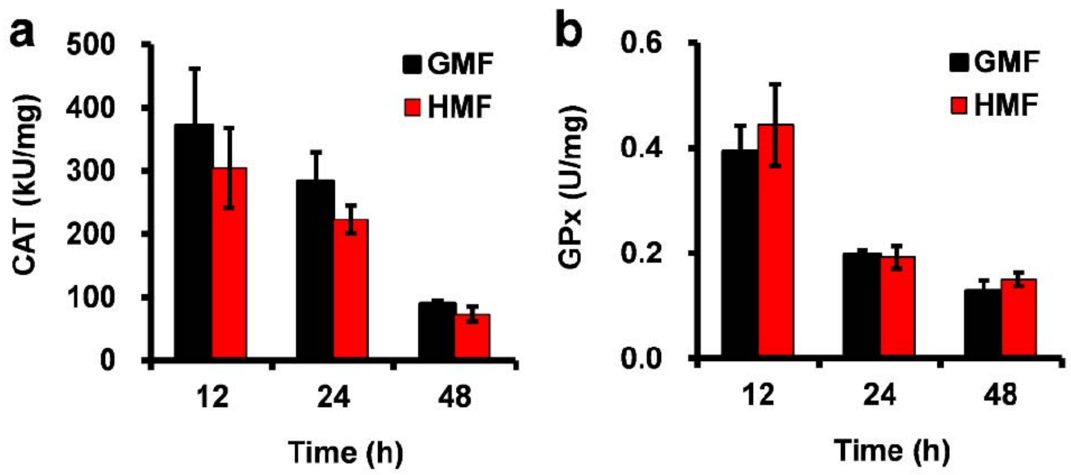

Supplementary Figure 3. The enzymatic degradation of H_2O_2 in the HMF. The activities of CAT (**a**) and GPx (**b**) were at the same levels between the HMF and GMF groups during the 48 h exposure period. Data were from independent experiments (n=3) and shown as mean ± s.e.m.

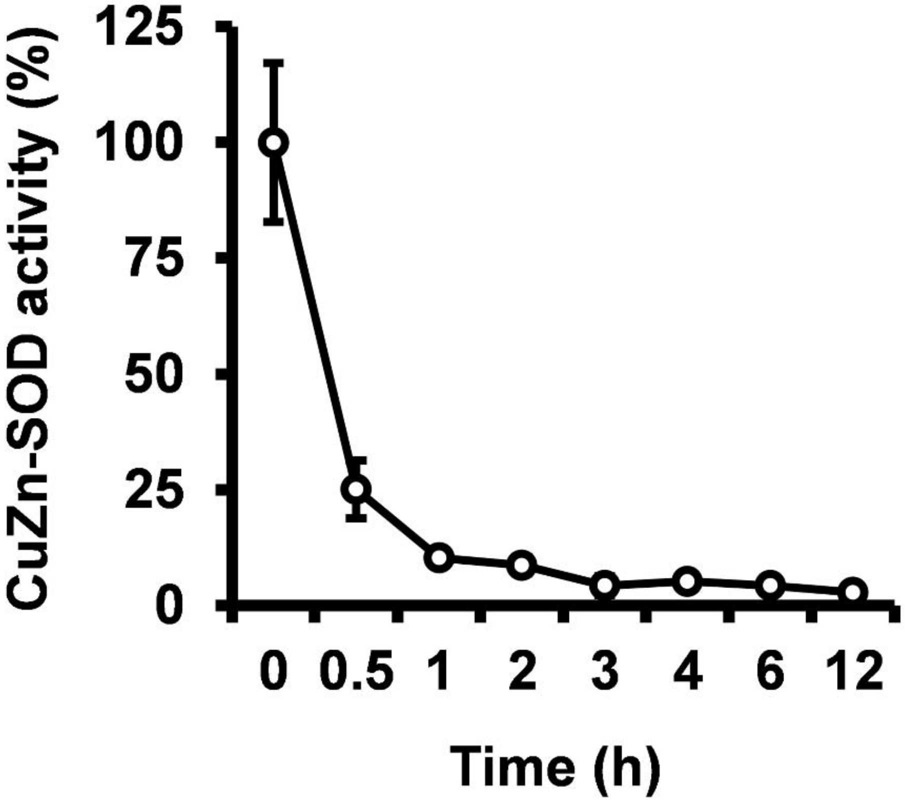

Supplementary Figure 4. The decrease of CuZn-SOD activity during thermal denaturation. The activity of CuZn-SOD decreased along with the incubation time, dropping down ~ 90% after 1 h incubation at 37 ℃. CuZn-SOD was dissolved in water. Data were from independent experiments (n=3) and shown as mean ± s.e.m.

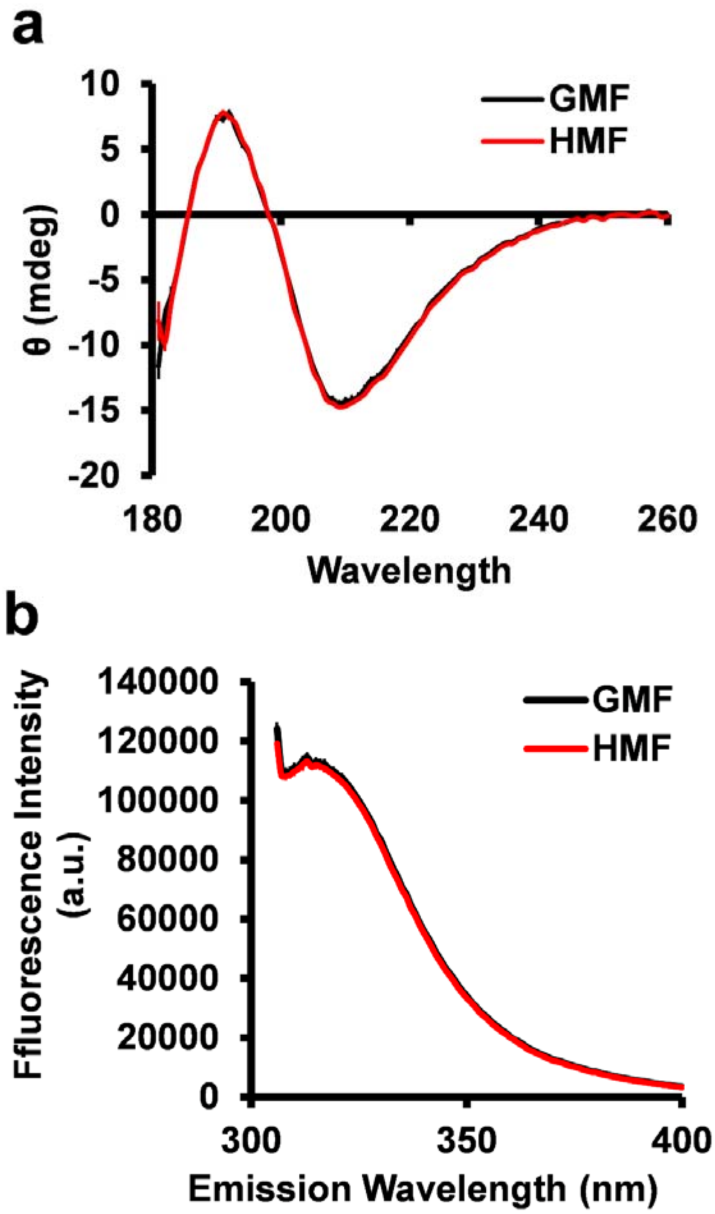

Supplementary Figure 5. The secondary structure and conformation of CuZn-SOD in the HMF. (**a**) CD assay showed that the secondary structure of CuZn-SOD was not changed after 0.5 h HMF exposure. (**b**) Intrinsic fluorescence assay indicated that the 0.5 h HMF exposure did not change the conformation of the enzyme. CuZn-SOD was dissolved in water. Data were from independent experiments (n=3) and shown as mean.
